# Abnormal amplitude of low‐frequency fluctuations and functional connectivity of resting‐state functional magnetic resonance imaging in patients with leukoaraiosis

**DOI:** 10.1002/brb3.714

**Published:** 2017-05-02

**Authors:** Rongchuan Cheng, Honglin Qi, Yong Liu, Shifu Zhao, Chuanming Li, Chen Liu, Jian Zheng

**Affiliations:** ^1^ Department of NeurologyThe Second Affiliated Hospital of the Third Military Medical UniversityChongqingChina; ^2^ Department of RadiologyThe First People's Hospital of Dadukou DistrictChongqingChina; ^3^ Department of RadiologyThe First Affiliated Hospital of the Third Military Medical UniversityChongqingChina

**Keywords:** amplitude of low‐frequency fluctuation, functional connectivity, leukoaraiosis, resting‐state functional magnetic resonance imaging, white matter hyperintensity

## Abstract

**Introduction:**

This study aimed to investigate the cerebral function deficits in patients with leukoaraiosis (LA) and the correlation with white matter hyperintensity (WMH) using functional MRI (fMRI) technology.

**Materials and Methods:**

Twenty‐eight patients with LA and 30 volunteers were enrolled in this study. All patients underwent structural MRI and resting‐state functional MRI (rs‐fMRI) scanning. The amplitude of low‐frequency fluctuations (ALFF) of rs‐fMRI signals for the two groups was compared using two‐sample t tests. A one‐sample *t* test was performed on the individual *z*‐value maps to identify the functional connectivity of each group. The *z* values were compared between the two groups using a two‐sample *t* test. Partial correlations between ALFF values and functional connectivity of the brain regions that showed group differences and Fazekas scores of the WMH were analyzed.

**Results:**

Compared with the control group, the LA group showed a significant decrease in the ALFF in the left parahippocampal gyrus (PHG) and an increased ALFF in the left inferior semi‐lunar lobule and right superior orbital frontal gyrus (SOFG). The patients with LA showed an increased functional connectivity between the right insular region and the right SOFG and between the right calcarine cortex and the left PHG. After the effects of age, gender, and years of education were corrected as covariates, the functional connectivity strength of the right insular and the right SOFG showed close correlations with the Fazekas scores.

**Conclusion:**

Our results enhance the understanding of the pathomechanism of LA. Leukoaraiosis is associated with widespread cerebral function deficits, which show a close correlation with WMH and can be measured by rs‐fMRI.

## 1 Introduction

Cognitive impairment is characterized by impaired cortical functions such as memory, orientation, computation, understanding, and visual spatial skills. The increasing prevalence of cognitive impairment with age seriously affects the health and lives of the elderly and is attracting more attention from research investigators (Hastings et al., 2014). Leukoaraiosis (LA) is the main pathological mechanism of vascular cognitive impairment (Longstreth et al., 2005; van Straaten et al., 2006). It commonly appears in subcortical atherosclerotic encephalopathy, chronic cerebral insufficiency, and other diseases and often affects the white matter in the periventricular and subcortical areas of the brain. The proportion of LA in patients who are aged 60 years and older is as high as 30%, as detected by brain magnetic resonance imaging (Launer et al., 2006; Nichtweiss, Weidauer, Treusch, & Hattingen, 2012).

According to the standards developed by Fazekas, Chawluk, Alavi, Hurtig, and Zimmerman (1987), the white matter hyperintensity (WMH) severity is assessed on fluid‐attenuated inversion recovery (FLAIR) images using a grading scale (grade 0, absent; grade 1, punctate; grade 2, early confluent; grade 3, confluent). Presently, the mechanism underlying WMH that affects brain function is not clear. With the development of neuroimaging, functional magnetic resonance imaging (fMRI) can study brain activity by detecting blood oxygen levels in the brain using high spatial resolution. fMRI includes task correlation and resting‐state fMRI (rs‐fMRI). Spontaneous neural activity can be detected in the brain, which is helpful for understanding the pathological mechanisms of nervous system diseases and mental diseases from the baseline level (He et al., 2007; Wu, Lai, Zhang, Yao, & Wen, 2015). Because rs‐fMRI is simple and easy to perform, is highly consistent, does not require task design, and facilitates patient cooperation, it has been commonly used in recent fMRI studies and is meaningful for clinical diagnosis and treatment evaluation. The amplitude of low‐frequency fluctuations (ALFFs) of rs‐fMRI can assess the amplitude of resting‐state spontaneous brain activity by calculating the square root of the power spectrum (typically in the frequency range of 0.01–0.08 Hz; Zang et al., 2007; Zou et al., 2008). Rs‐fMRI has been proven effective for reflecting the spontaneous neural activity in both animals and humans (Goncalves et al., 2006; Leopold, Murayama, & Logothetis, 2003). Our hypothesis is as follows: (1) changes in intrinsic brain activity patterns in patients with LA could be located in multiple brain regions; and (2) the features of ALFF and functional connectivity in specific regions correlate with the disease severity of LA.

## 2 Materials and Methods

### 2.1 Subjects

This study was approved by the Medical Ethics Committee at our institution. Informed consent was obtained from each subject. Twenty‐eight patients diagnosed with brain ischemic diseases such as subcortical arteriosclerotic encephalopathy or chronic cerebral circulation insufficiency were recruited from the Neurology Department of our hospital (Table 1). All patients had subcortical WMH on FLAIR images from conventional MRI. The exclusion criteria included cerebral hemorrhages, neurodegenerative diseases, infarcts, sarcoidosis, multiple sclerosis, brain irradiation, or normal pressure hydrocephalus. All patients completed formal neuropsychological assessments using the following tests: the Boston Naming Test, verbal and categorical fluency test, figural recognition test, auditory verbal learning test, Clinical Dementia Rating, the Montreal Cognitive Assessment (MoCA), and the Mini‐Mental State Examination (MMSE). Patients with severe claustrophobia, a Hamilton Depression Rating Scale score ≥18 and contraindications to MRI were also excluded. Thirty healthy elderly subjects without known nervous system diseases, neurological or psychiatric disorders, or vascular risk factors were recruited as healthy controls.

**Table 1:** Demographic and clinical data of patients with leukoaraiosis and control subjects

Characteristics	Patients with leukoaraiosis (*n *=* *28)	Control subjects (*n *=* *30)	*p* Value
Gender (male/female)	16/12	14/16	.45^a^
Age (years)	67.9 ± 6.1	66.6 ± 4.6	.38^b^
Education (years)	10.1 ± 3.4	11.0 ± 4.2	.42^b^
MMSE	27.89 ± 1.57	28.10 ± 1.73	.73^b^
MoCA	25.71 ± 2.00	25.93 ± 1.80	.71^b^
Fazekas scores	2.89 ± 1.17	—	
Mean FD (mm)	0.13 ± 0.04	0.12 ± 0.04	.58

MMSE, Mini‐Mental State Examination; MoCA, Montreal Cognitive Assessment; FD, framewise displacement.

a *p* Values for gender distribution between the two groups was obtained using a two‐tailed chi‐squared test.

b *p* Values were obtained using a two‐sample two‐tailed *t* test.

### 2.2 Scan acquisition

The MRI data for all patients were obtained using a SIEMENS Trio 3‐Tesla scanner (Siemens, Erlangen, Germany). First, a conventional transverse FLAIR sequence was used (TR = 9,000 ms, TE = 93 ms, TI = 2,500 ms, flip angle = 130°, thickness = 4.0 mm, matrix = 256 × 256, voxel size = 0.9 × 0.9 × 4 mm^3^). Then, the functional images were scanned using an echo‐planar imaging sequence (TR = 2,000 ms, TE = 30 ms, flip angle = 90°, thickness = 3.0 mm, matrix = 64 × 64, voxel size = 3.5 × 3.5 × 3.0 mm^3^). Finally, three‐dimensional T1‐weighted magnetization‐prepared rapid gradient‐echo sagittal images were acquired with the following parameters: TR = 1,900 ms, TE = 2.52 ms, TI = 900 ms, thickness = 1.0 mm, flip angle = 9°, matrix = 256 × 256, voxel size = 1 × 1×1 mm^3^.

### 2.3 Image processing and analysis

The structural data were obtained with an optimized VBM8 protocol (http://dbm.neuro.uni-jena.de/vbm/) for the statistical parametric mapping package (SPM8, www.fil.ion.ucl.ac.uk/spm). The structural images were smoothed to a Gaussian kernel of 6‐mm full width at half maximum (FWHM). After motion correction, all structural images were coregistered with the mean rs‐fMRI image, corrected for bias‐field inhomogeneity, and registered using linear and nonlinear transformations. Finally, the images were segmented into gray matter, white matter, and cerebrospinal fluid. The WMH severity was evaluated according to the grading scale presented by Fazekas on FLAIR images based on the agreement of two experienced neurologists.

SPM8 and a toolbox, namely, Data Processing & Analysis for Brain Imaging (http://rfmri.org/dpabi) (Yan, Wang, Zuo, & Zang, 2016) software were used to process the functional data. For all subjects, the first five volumes of each time series were discarded, and the remaining 235 volumes were corrected for the within‐scan acquisition time differences between slices and were further realigned to the first volume. Head movement parameters were calculated, and subjects with more than 2° of head rotation or 2 mm of displacement were excluded. Two‐sample *t* tests were used to compare the group differences in head motion using the means of the framewise displacement (FD) Jenkinson measurement. Functional volumes were spatially normalized to the Montreal Neurological Institute space and smoothed with a 6‐mm FWHM Gaussian kernel.

REST software (www.Restfmri.net) was used to calculate the ALFF. The functional time series of all subjects were transformed to the frequency domain using a fast Fourier transform algorithm. The time series for each voxel was band‐pass filtered (0.01–0.08 Hz). The averaged square root was termed the ALFF. Finally, the original ALFF value for each voxel was divided by the global mean ALFF value to standardize data across subjects.

A seed voxel correlation approach was used to examine the functional connectivity of all subjects. Brain regions that showed ALFF alterations for patients with LA were selected as regions of interest (ROIs), and a reference time series was extracted by averaging the fMRI time series of all voxels within the ROIs. Correlations were computed among each seed reference and the rest of the brain in a voxel‐wise manner. The correlation coefficients were transformed to *z* values using Fisher's *r*‐to‐*z* transformation. Components with a high correlation with cerebrospinal fluid and white matter or with a low correlation with gray matter were removed.

### 2.4 Statistical analysis

Amplitude of low‐frequency fluctuation comparisons between patients with LA and healthy controls were performed using two‐sample *t* tests. A one‐sample *t* test was performed on the individual *z*‐value maps to examine functional connectivity of each group. The two‐sample *t* test was used to compare the *z* values in each voxel of the two groups. We considered the whole brain gray matter volume, mean FD, age, gender, and education years as covariates. The relationship between functional connectivity and Fazekas scale scores was analyzed by partial correlation. The significance was set at *p *<* *.05 corrected with a multiple comparison correction.

## 3 Results

The patient and healthy control groups were not significantly different in age, gender, education levels, or mean FD (Table 1). There was no significant difference between the MMSE and MoCA scores of the two groups (*t *=* *0.71–0.73, *p* < .001; Table 1).

**Figure 1:**
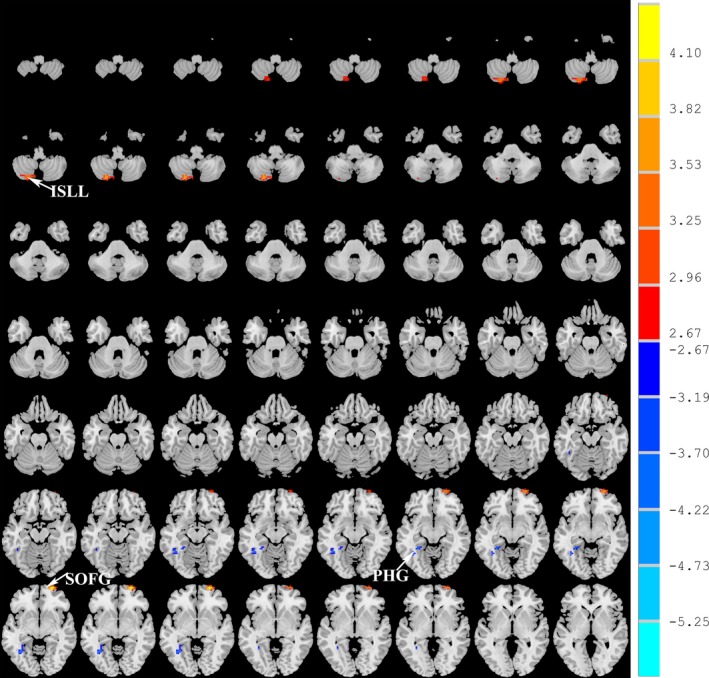
Brain regions with abnormal amplitude of low‐frequency fluctuations (ALFF) in leukoaraiosis patients are shown. These regions are described in detail in Table 2. Statistical thresholds were set at *p* < .01 for individual voxels and a cluster size >1,080 mm^3^, which corresponds to a corrected *p* < .01 as determined by Monte Carlo simulations. Color bars represent the *t* value of the group analysis. A cool color represents decreased ALFF values, and a warm color represents increased ALFF values. PHG, parahippocampal; ISLL, inferior semi‐lunar lobule; SOFG, frontal superior orbital gyrus

**Figure 2:**
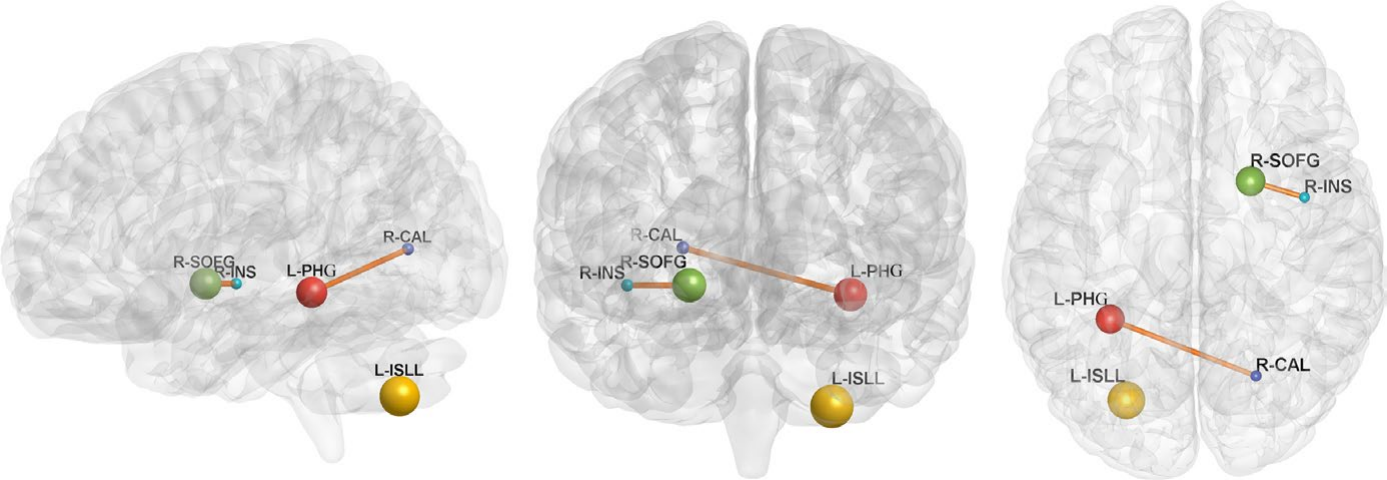
Brain functional connectivity alterations of leukoaraiosis patients are shown. Increased functional connectivity was found among the right insular and the right SOFG, the right calcarine cortex and the left PHG (*p* < .05, FDR corrected). Brain graph created using BrainNet Viewer (http://www.nitrc.org/projects/bnv/). The brain network edges were extracted from the correlation matrix of rs‐fMRI connectivity across the regions of interest. Ball locations represent the peak Montreal Neurological Institute Coordinate System coordinate of differences within clusters, and the size indicates the cluster size. PHG, parahippocampal; ISLL, inferior semi‐lunar lobule; SOFG, frontal superior orbital gyrus; INS, insula; CAL, calcarine

**Figure 3:**
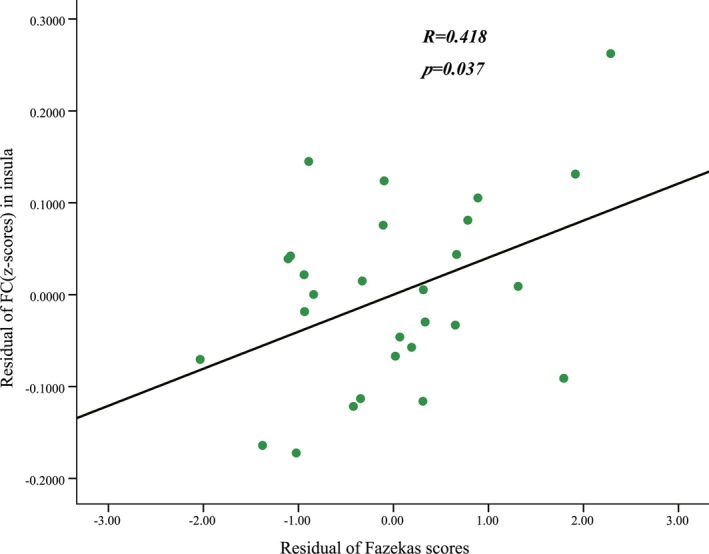
Correlations between the functional connectivity strength of the left insular and the right SOFG with the Fazekas scores. The effects of age, gender, and years of education were corrected as covariates. Each dot represents the data from one study participant. SOFG, frontal superior orbital gyrus

Compared with normal volunteers, the patients with LA showed significantly decreased ALFF in the left parahippocampal gyrus (PHG) and increased ALFF in the left inferior semi‐lunar lobule (ISLL) and right frontal superior orbital gyrus (SOFG) (*p *<* *.05, FWE corrected; Figure 1, Table 2). Brain regions that showed group ALFF differences were selected as seed regions. The patients with LA showed an increased connectivity between the right insular region and the right SOFG, as well as between the right calcarine cortex and the left PHG (Figure 2). After the effects of age, gender, and years of education were corrected as covariates, the functional connectivity strength of the right insular and the right SOFG showed closed correlations with the Fazekas scores (Figure 3).

**Table 2:** Regions showing ALFF value differences between patients with leukoaraiosis and control subjects

Brain regions	Number of cluster voxels	MNI coordinate (mm)			Maximum *t*
		*x*	*y*	*z*	
Decreased ALFF					
Left PHG	40	−30	−45	−9	−3.86
Increased ALFF					
Left ISLL	53	−24	−75	−45	3.80
Right SOFG	41	22	6	−6	4.39

Comparisons were performed at *p *<* *.01 and corrected for multiple comparisons using the AlphaSim program. *x*,* y*, and *z* are coordinates of peak locations in the MNI space. Maximum *t* shows ALFF differences between the leukoaraiosis group and healthy subjects. A positive maximum *t*‐score represents an increase, and a negative maximum *t*‐score represents a decrease. ALFF, amplitude of low‐frequency fluctuations; MNI, Montreal Neurological Institute Coordinate System or Template; PHG, parahippocampal; ISLL, inferior semi‐lunar lobule; SOFG, frontal superior orbital gyrus.

## 4 Discussion

To date, given its better availability, noninvasiveness, and lack of radiation exposure, fMRI has been used as an effective technique for the study of central nervous system disorders (Baudrexel et al., 2011; Hacker, Perlmutter, Criswell, Ances, & Snyder, 2012; Helmich et al., 2010; Kwak et al., 2010, 2012; Skidmore et al., 2013; Tessitore et al., 2012; Wu et al., 2009, 2011). Rs‐fMRI can investigate the intrinsic spontaneous brain neural activity during rest without task performance. Most rs‐fMRI studies have focused on amplitude of low‐frequency oscillations at a frequency band of 0.01–0.08 Hz of the BOLD signal (Logothetis, Pauls, Augath, Trinath, & Oeltermann, 2001; Qi et al., 2010; Raichle et al., 2001; Yang et al., 2007). Both animal and human studies have provided evidence that fMRI effectively identifies cortical spontaneous neuronal function (Moosmann et al., 2003; Pelled & Goelman, 2004).

In this study, we used rs‐fMRI to systematically investigate the changes in the ALFF of intrinsic brain activity in patients with LA. Reduced ALFF was observed in the left PHG, and increased ALFF was observed in the left ISLL and right SOFG. These findings suggest that the left PHG, left ISLL, and right SOFG may be the areas most affected by ischemic WMH. The altered spontaneous ALFF values in these areas are possible characteristics of the neurological impairments of LA. In behavioral studies, LA patients have been found to have significant cognitive impairments of attention, memory, executive function, and information processing speed (Defrancesco et al., 2013; Yuan et al., 2012). The PHG is considered to be critical for memory function, including memory encoding and retrieval. It is also closely associated with human learning, emotions, and other activities. Previous studies have found that the hippocampus and PHG are susceptible to ischemia and lower blood volume (Chai, Ofen, Jacobs, & Gabrieli, 2010; Fein et al., 2000; Yang et al., 2010). The reduced ALFF activities in the PHG may indicate memory impairment in human subjects who have LA. The ISLL is part of the cerebellum, which has not been commonly reported in previous fMRI investigations. In traditional views, the cerebellum functions focus on control of motor behavior and coordination. Recently, the lateral cerebellum has been discovered to play an important role in memory function (Jantzen, Oullier, Marshall, Steinberg, & Kelso, 2007). The relationship between memory load and BOLD signal changes in the inferior cerebellum has also been proven (Kirschen, Chen, & Desmond, 2010; Ng et al., 2016; Tamagni et al., 2010). The SOFG has core components of the emotional processing network and is involved in the perceptual processing of emotions in facial expressions (Leppanen & Nelson, 2009). Furthermore, it has been linked to the experience of anger and aggression (Lindquist, Wager, Kober, Bliss‐Moreau, & Barrett, 2012; Vytal & Hamann, 2010). Given that the SOFG is associated with negative emotion processing, the increased ALFF in these areas suggests that people with LA may experience more negative emotions. This speculation is in accordance with the finding that impaired white matter is linked to anxiety and depression (Coplan et al., 2010).

In addition to regional ALFF, rs‐fMRI can provide information about functional connectivity. The analysis of cross correlations between the ALFF of spatially remote regions can examine the brain connectivity and networks. This is the first study to investigate the brain functional connectivity alterations in patients with LA. The patients with LA showed an increased connectivity between the right SOFG and the right insula, which also showed closed correlations with the Fazekas scores. The human insular has an important role in attention, language, speech, working memory, and memory (Kelly et al., 2012). The increased functional connectivity between insula and SOFG was earlier reported in the Alzheimer's Disease(Balthazar et al., 2014; Hafkemeijer et al., 2017). Insula and SOFG play an important role in anterior frontoinsular‐cingulo‐orbitofrontal network often called the salience network which is related with the ability to direct attentional resources and goal‐relevant cognition (Menon, 2011). The Fazekas score was designed for the cross‐sectional rating of WMH. It is stable and reliable and has strong ties to WMH severity (Kapeller et al., 2003). The calcarine was suggested to be a key hub of the posterior compensatory network in cognitively proficient elderly with hippocampal atrophy in a spatial working memory task fMRI(Valenzuela et al., 2015). PHG is considered as the hub of the default mode network and plays a mediation role to convey information to the frontal cortex, where information is integrated. The disrupted functional connectivity between CAL and PHG has also been founded in MCI in functional brain network study (Lou et al., 2016). Previously, increased connectivity was observed in other diseases. Patients with restless legs syndrome showed significantly increased connectivity in the sensory thalamic, ventral and dorsal attention, basal ganglia‐thalamic, and cingulate networks (Gorges et al., 2016). Patients with mild cognitive impairment showed increased connectivity in the default mode network and visual network (Cai et al., 2016). The precise mechanisms leading to the increased connectivity in LA are not clearly.

In summary, in this study, we examined the LA ‐related cerebral function deficits of the ALFF during the resting state. We found that several cerebral regions were significantly correlated with WMH, including the left PHG, the ISLL, and the SOFG. The patients with LA showed an increased connectivity between the right SOFG and the right insular, which was closely correlated with Fazekas scores and suggests a compensatory mechanism. These findings may contribute to our understanding of the mechanism of LA. The main limitation of this study is the relatively small sample size of patients with LA. Additionally, the alterations of BOLD signal may be derived from both neural and noise contributions (Di, Kim, Huang, Lin, & Biswal, 2013), although we employed a range of data preprocessing steps to mitigate the side effects. Furthermore, we could not observe dynamic ALFF changes in different stages of LA due to the cross‐sectional group data. Future studies should address these issues through longitudinal evaluations of a large sample of patients with LA.

## Conflict of Interest

Rongchuan Cheng, Honglin Qi, Yong Liu, Shifu Zhao, Chuanming Li, Chen Liu, and Jian Zheng declare that they have no conflict of interest.
